# Increased α2-6 sialylation of endometrial cells contributes to the development of endometriosis

**DOI:** 10.1038/s12276-018-0167-1

**Published:** 2018-12-12

**Authors:** Hee-Jin Choi, Tae-Wook Chung, Hee-Jung Choi, Jung Ho Han, Jung-Hye Choi, Cheorl-Ho Kim, Ki-Tae Ha

**Affiliations:** 1 Department of Korean Medical Science, School of Korean Medicine, Seoul, Republic of Korea; 2 Healthy Aging Korean Medical Research Center, Pusan National University, Yangsan, Gyeongnam 50612 Republic of Korea; 3 Graduate Training Program of Korean Medicine for Healthy-aging, Pusan National University, Yangsan, Gyeongnam 50612 Republic of Korea; 4 Department of Life and Nanopharmaceutical Sciences and Department of Oriental Pharmacy, Kyung Hee University, Seoul, 02447 Republic of Korea; 5 Department of Biological Science, Sungkyunkwan University, Suwon, Kyunggi-do 16419 Republic of Korea

## Abstract

Endometriosis is a disease characterized by implants of endometrial tissue outside the uterine cavity and is strongly associated with infertility. Focal adhesion of endometrial tissue to the peritoneum is an indication of incipient endometriosis. In this study, we examined the effect of various cytokines that are known to be involved in the pathology of endometriosis on endometrial cell adhesion. Among the investigated cytokines, transforming growth factor-β1 (TGF-β1) increased adhesion of endometrial cells to the mesothelium through induction of α2-6 sialylation. The expression levels of β-galactoside α2-6 sialyltransferase (ST6Gal) 1 and ST6Gal2 were increased through activation of TGF-βRI/SMAD2/3 signaling in endometrial cells. In addition, we discovered that terminal sialic acid glycan epitopes of endometrial cells engage with sialic acid-binding immunoglobulin-like lectin-9 expressed on mesothelial cell surfaces. Interestingly, in an in vivo mouse endometriosis model, inhibition of endogenous sialic acid binding by a NeuAcα2-6Galβ1-4GlcNAc injection diminished TGF-β1-induced formation of endometriosis lesions. Based on these results, we suggest that increased sialylation of endometrial cells by TGF-β1 promotes the attachment of endometrium to the peritoneum, encouraging endometriosis outbreaks.

## Endometriosis: a sweet and sticky mechanism uncovered

A growth factor involved in cell differentiation and proliferation contributes to the development of endometriosis by stimulating a protein modification mechanism that increases the adhesiveness of cells lining the uterus. Endometriosis results when these cells, known as endometrial cells, start growing outside the uterus causing pelvic pain, heavy periods and, in some cases, infertility. Ki-Tae Ha at Pusan National University, Yangsan, South Korea, and colleagues found that transforming growth factor-β1 signaling promoted the addition of sialic acid sugar units onto endometrial cell surface proteins. This modification enhanced the adhesion of endometrial cells to mesothelial cells, which line other internal organs, and the formation of endometriosis lesions in mice. Preventing sialic acid binding to its mesothelial cell receptor reduced lesion formation. The findings reveal a new molecular mechanism underlying endometriosis and a potential treatment strategy.

## Introduction

Endometriosis is a common chronic gynecological disorder that affects ~ 10% of women of reproductive age^1^. It is characterized by the presence of endometrial tissue outside the uterus and is associated with pelvic pain, dysmenorrhea, and infertility^2^. Surgical treatment aims to remove the endometriotic lesions, and medical follow-up monitors and controls symptoms and recurrence. However, conventional therapy cannot efficiently reduce the high relapse rate of endometriosis^3^. Despite the fact that endometriosis is a significant disease in fertile women—because of its association with infertility—the molecular mechanisms remain unclear^4^. Therefore, more research examining the factors related to endometriosis recurrence is needed for managing endometriosis.

The theory of retrograde menstruation suggests that reflux of endometrial tissues during menstruation is the source of ectopic endometrium, and it is the most widely accepted hypothesis of pathogenesis in endometriosis^2^. At the initial stage of the disorder, adhesion of refluxed endometrial tissues to the peritoneal mesothelium is critical in ectopic endometriosis lesion formation^5^. In women suffering from endometriosis, modified expression of cytokines and growth factors creates a microenvironment that promotes adhesion of the endometrium to the peritoneum^6^. A number of cytokines, such as transforming growth factor-β1 (TGF-β1), tumor necrosis factor alpha (TNF-α), interferon gamma (INF-γ), interleukin (IL)-1β, IL-6, and IL-8, have been suggested to induce the expression of adhesion molecules on the surface of human endometrial cells^7–9^. In this respect, investigating and regulating the mechanism of cytokine-induced endometrial cell attachment may be an effective method for preventing endometriosis relapse.

Although endometriosis is a benign disorder, it exhibits characteristics similar to those of cancer, such as cell proliferation, migration, invasion, and adhesion^6^. Glycosylation is one of the most common post-translational modifications of secretory and membrane proteins in all eukaryotes and modulates cell–cell and cell–microenvironment interactions^10,11^. Aberrant sialylation promotes cancer cell metastasis by increasing adhesion of cancer cells to the extracellular matrix^12,13^. Similarly, it has been reported that the levels of glycoproteins are increased in serum, peritoneal fluid, and eutopic endometrium of patients with endometriosis^14–16^. Moreover, inhibition of CD44 glycosylation decreases attachment of endometrial cells in early endometriotic lesion establishment^17^. However, the effect and underlying mechanisms of altered sialylation on endometriosis establishment, especially on the adhesion between endometrial cells and peritoneal mesothelial cells, are still unclear.

In the present study, we demonstrated the effect of sialylation on the adhesion of endometrial cells and found that TGF-β1 increased the adhesion of endometrial cells to peritoneal mesothelial cells through induction of α2-6 sialylation. We also determined that sialic acid epitopes of endometrial cells interacted with sialic acid-binding immunoglobulin-like lectin (Siglec)-9 expressed in peritoneal mesothelial cells. Furthermore, inhibition of glycan binding prevented the formation of TGF-β1-induced endometriotic lesions in a mouse endometriosis model. Therefore, we suggest that altered sialylation of endometrial cells plays a pivotal role in the initiation of endometriosis.

## Materials and methods

### Antibodies and reagents

Recombinant human TGF-β1 (100–21), IL-1β (200-01B), IL-6 (200–06), and IL-8 (200–08 M) cytokines were purchased from Peprotech (Rocky Hill, NJ). Cell Tracker™ Green CMFDA (5-chloromethylfluorescein diacetate) was supplied by Thermo Fisher Scientific (Waltham, MA). α2–3,6,8 Neuraminidase (P0720S) was acquired from New England Biolabs (Ipswich, MA). Biotinylated *Maackia amurensis* lectin II (MAL II) and biotinylated *Sambucus nigra* lectin (SNA) were provided by Vector Laboratories (Burlingame, CA). NeuAcα2–3Galβ1-4GlcNAc (3ʹ-SLN) and NeuAcα2-6Galβ1-4GlcNAc (6ʹ-SLN) were purchased from Carbosynth (Berkshire, UK). TGF-βRI inhibitor (SB525334) was purchased from Sigma-Aldrich (St. Louis, MO), and cells were treated with 10 μm SB525334 1 h before TGF-β1 (10 ng/mL) stimulation. Information regarding the antibodies used in this study is listed in Supplementary Table 1.

### Cell culture

Immortalized human endometriotic epithelial cells (12Z cells)^18^ were generously provided by Dr. Starzinski-Powitz (Johann-Wolfgang-Goethe-Universitaet, Germany). Human endometrial cells derived from human adenocarcinoma (Ishikawa cells)^19^ were established by Dr. Nishida (National Hospital Organization, Kasumigaura Medical Center, Japan) and were generously provided by Dr. Jacques Simard (CHUL Research Center, Quebec, Canada). Immortalized human endometrial stromal cells (THESCs) and human peritoneal mesothelial cells (Met-5A cells) were purchased from American Type Culture Collection (ATCC; Rockville, MD).

Ishikawa cells were grown in Dulbecco’s modified Eagle’s medium (Welgene, Daegu, Republic of Korea), and 12Z cells were maintained in Roswell Park Memorial Institute medium (1640; Lonza, Basel, Switzerland). Both media were supplemented with 10% fetal bovine serum (FBS; Thermo Fisher Scientific), 100 U/mL penicillin and 100 μg/mL streptomycin (Thermo Fisher Scientific), and cells were cultured at 37 °C in a humidified atmosphere with 5% CO_2._ Met-5A cells were cultured in Medium 199 (M199; Welgene) containing 10% FBS, 100 U/mL penicillin and 100 μg/mL streptomycin. THESCs were grown in 1:1 mixture of Dulbecco’s modified Eagle’s medium and Ham’s F-12 medium without phenol red supplemented with 100 U/mL penicillin, 100 μg/mL streptomycin, 1% ITS + Premix (BD Biosciences; San Jose, CA), 500 ng/mL puromycin, and 10% charcoal/dextran (Sigma-Aldrich)-treated FBS.

### Cell adhesion assay

Met-5A cells were seeded on six-well plates and cultured to a confluent monolayer. THESCs, Ishikawa, and 12Z cells were incubated in serum-free medium containing human recombinant cytokines for 48 h. THESCs, Ishikawa, and 12Z cells were labeled with Cell Tracker™ Green CMFDA according to the manufacturer’s protocol. In brief, we aspirated the supernatant and added pre-warmed Cell Tracker™ working solution (5 μm CMFDA in serum-free medium). After 20 min of incubation under growth conditions, we removed the Cell Tracker™ working solution and washed the cells three times. Cells were harvested by centrifugation and resuspended in culture medium. Fluorescence-labeled cells were added onto a monolayer of Met-5A cells. After incubation for 20 min at 37 °C, the cells were washed with phosphate-buffered saline (PBS) three times to remove non-binding cells. Adhered cells were visualized using a fluorescence microscope (Axio Imager M1, Zeiss, Germany) by measuring the excitation at 470 nm and emission at 525 nm. Five fields in each sample were chosen randomly, and the number of adhered cells were averaged after quantification with ImageJ software (NIH; Bethesda, MD).

### Lectin blot analysis

Cells were homogenized in radioimmunoprecipitation assay buffer (RIPA buffer) (20 mm Tris-HCl/pH 7.5, 1% NP-40, 150 mm NaCl, 1 mm EDTA, 1 mm EGTA, 1 mm Na_3_VO_4_, 1% sodium deoxycholate, 2.5 mm sodium pyrophosphate, 1 mm β-glycerophosphate, and 1 μg/mL leupeptin), and the concentration of extracted proteins was determined with a Bradford assay. Equal amounts (10 μg) of proteins were separated using sodium dodecyl sulfate polyacrylamide gel electrophoresis (SDS-PAGE) and transferred onto a nitrocellulose membrane (GE Healthcare Life Sciences; Buckinghamshire, UK). The membranes were blocked for 30 min in Carbo-free blocking solution (Vector Laboratories) prior to incubation with 10 μg/mL MAL II and SNA at room temperature for 1 h. After incubation of the membranes with Vectastain (Vector Laboratories) at room temperature for 30 min, the proteins of interest were visualized using a Chemiluminescent/Fluorescent Substrate Kit (Vector Laboratories).

### Lectin fluorescence-activated cell sorting (FACS) analysis

THESCs, Ishikawa, and 12Z cells were fixed with 3.7% formaldehyde and incubated with 10 μg/mL biotinylated lectins, MAL II and SNA for 2 h at 4 °C. After incubation with Alexa Fluor 488-conjugated streptavidin (Thermo Fisher Scientific) for 30 min at 4 °C, the cells were evaluated by flow cytometry (BD FACSCANTO II; BD Biosciences) at an excitation wavelength of 488 nm and an emission wavelength of 530 nm.

### Reverse transcription-polymerase chain reaction (RT-PCR)

Total RNA was isolated from cells using a GeneJET RNA Purification Kit (Thermo Fisher Scientific) according to the manufacturer’s instructions. Synthesis of complementary DNA was carried out using RevertAid Reverse Transcriptase (Thermo Fisher Scientific), and targeted genes were amplified using AccuPower® PCR PreMix (Bioneer; Daejon, Republic of Korea). The sequences of the primers used in this study are listed in Supplementary Table 2.

### Western blot analysis

Total protein was isolated in RIPA buffer. Extracted protein was separated on a 10% SDS-PAGE gel, and size-fractioned protein samples were transferred to a nitrocellulose membrane. Membranes were blocked with 5% skim milk (Sigma-Aldrich) and incubated with primary antibodies overnight at 4 °C. After reaction with the appropriate horseradish peroxidase-conjugated secondary antibodies, signaling was visualized using an ECL chemiluminescence system (Thermo Fisher Scientific).

### Gene knockdown with small interfering RNA (siRNA)

To knock down endogenous human ST6Gal1, ST6Gal2, and Siglec-9, human siRNA constructs were obtained from Bioneer. Ishikawa and Met-5A cells (5 × 10^5^ cells/well) were seeded into six-well plates. Cells were transfected with siRNAs (200 nm) using Lipofectamine® RNAiMAX Reagent (Thermo Fisher Scientific) according to the manufacturer’s protocol. The knockdown efficiency of siRNA was verified by RT-PCR and western blot analysis 24 h after transfection.

### Animals

Six-week-old female C57BL/6 mice were purchased from Orient Bio (Seongnam, Republic of Korea). The mice were ovariectomized and recuperated for 14 days before estrogen treatment. Animals were fed a mouse diet (Orent Bio) with ad libitum access to water and maintained under controlled conditions with a light/dark cycle of 12:12 h. All experimental procedures described here were approved by the Institutional Animal Care and Use Committee of Pusan National University (Pusan, Republic of Korea; PNU-2017-1430).

### Induction of the in vivo endometriosis model

Endometriosis lesions were induced according to the method described by Somigliana et al.^20^. Both donor and recipient mice were subjected to an ovariectomy 14 days before receiving estrogen treatment and injected subcutaneously with 100 mg/kg β-estradiol (Santa Cruz Biotechnology; Dallas, TX) in corn oil every week, starting from 7 days before endometriosis induction. One donor mouse was sacrificed for every two mice challenged with endometriosis. Uterine sa.mples to be inoculated were isolated from syngeneic donor mice and finely chopped. Endometrial fragments were suspended in 0.8 mL of PBS and used to inoculate the peritoneal cavity of recipient mice at day 0. Mice challenged with endometriosis were injected intraperitoneally with 2.5 μg/kg recombinant mouse TGF-β1 (Peprotech) at days 0, + 2, and + 4. 6ʹ-SLN (1 or 2 mg/kg) was administered intraperitoneally as a daily injection for 21 days starting from endometriosis induction. Three weeks after endometrium challenge, the mice were killed, and endometriotic lesions were excised from the surrounding tissue to evaluate number, weight and volume (*n* = 6 mice per group).

### Statistical analysis

The number of adhered endometrial cells and the expression levels of sialylation were converted into fold differences compared with the control group. The values are expressed as the mean ± standard deviation. A two-tailed Student’s *t* test for comparison between two different groups or one-way ANOVA followed by Tukey’s ad hoc test for comparison between multiple groups was performed with the assistance of GraphPad Prism software (GraphPad Software, San Diego, CA, USA). The minimum significance level was set at a *p* value of 0.05 for all analyses. All experiments were independently conducted three times, except the animal study.

## Results

### TGF-β1 increases the adhesion of endometrial cells to peritoneal mesothelial cells through induction of sialylation in endometrial cells

**Fig. 1:**
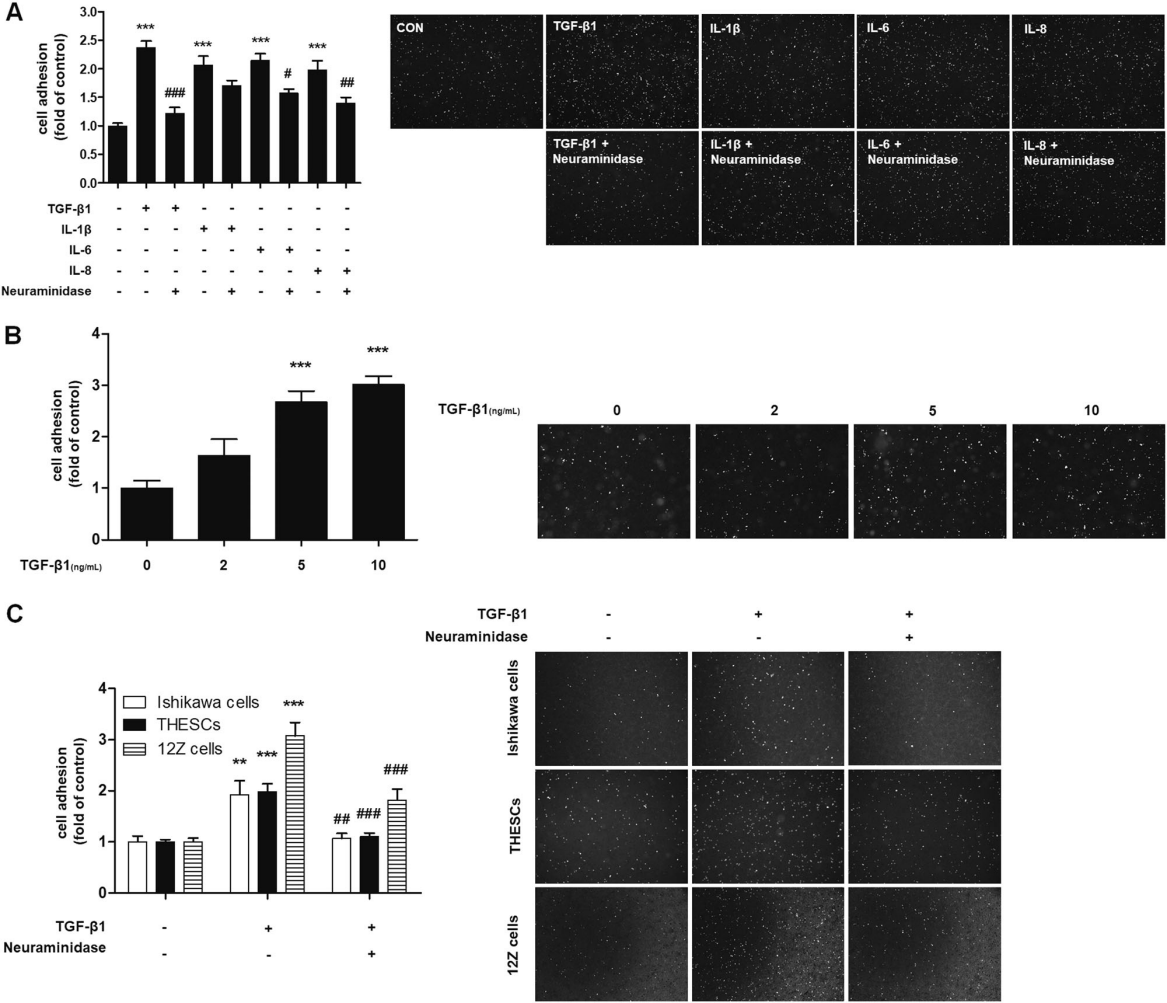
TGF-β1 enhances the adhesion of endometrial cells by increasing sialylation on *N*-glycoproteins **a–c** Fluorescence-labeled endometrial (Ishikawa or THESCs) or endometriotic (12Z) cells were added to a confluent monolayer of Met-5A cells. After 20 min of incubation at 37 °C, the number of adhered Ishikawa, THESCs or 12Z cells was calculated and is expressed as fold-difference relative to the control (mean ± SD). **a** Ishikawa cells were treated with neuraminidase (4 U/mL) for 1 h and then stimulated with cytokines (10 ng/mL) for 48 h. **b** Ishikawa cells were stimulated with the indicated concentration of TGF-β1 for 48 h. **c** Ishikawa, THESCs and 12Z cells were incubated with neuraminidase 1 h before TGF-β1 (10 ng/mL) stimulation. * *p* < 0.05, ** *p* < 0.01, and *** *p* < 0.001 compared with the control group. ^#^ *p* < 0.01 compared with the TGF-β1-treated group

To investigate the effects of cytokines on endometrial cell adhesion to mesothelial cells, before the adhesion assay, Ishikawa cells were treated with six types of cytokines known to be associated with endometrial cell attachment^7–9^. Among them, TGF-β1, IL-1β, IL-6, and IL-8 treatment increased the adhesion of Ishikawa cells to Met-5A cells (Fig. 1a). On the other hand, TNF-α and INF-γ treatment decreased the adhesion of Ishikawa cells compared with untreated cells (Supplementary Figure 1).

It has been reported that aberrant sialylation on cancer cell surfaces plays a vital role in the regulation of adhesion between cells and the extracellular matrix^21^. Therefore, we determined the role of sialylation in endometrial cell attachment by using neuraminidase—a glycoside hydrolase enzyme that cleaves the glycosidic linkage of sialic acid—before cytokine stimulation. As shown in Fig. 1a, neuraminidase treatment abolished cytokine-induced adhesion of endometrial cells, especially in TGF-β1-treated Ishikawa cells. As the adhesion of TGF-β1-treated Ishikawa cells showed the highest sialic acid dependence, further studies were performed with TGF-β1-stimulated Ishikawa cells. As shown in Fig. 1b, TGF-β1 treatment elevated cell adhesion levels in Ishikawa cells in a concentration-dependent manner. Similarly, TGF-β1 also increased the adhesion of THESCs and 12Z cells to Met-5A cells, whereas neuraminidase treatment alleviated the adhesion (Fig. 1c). These results suggest that TGF-β1 enhances the adhesion of endometrial cells to peritoneal mesothelial cells through sialylation of endometrial cells.

### TGF-β1 induces α2-6 sialylation by enhancing sialyltransferase expression in endometrial cells

**Fig. 2:**
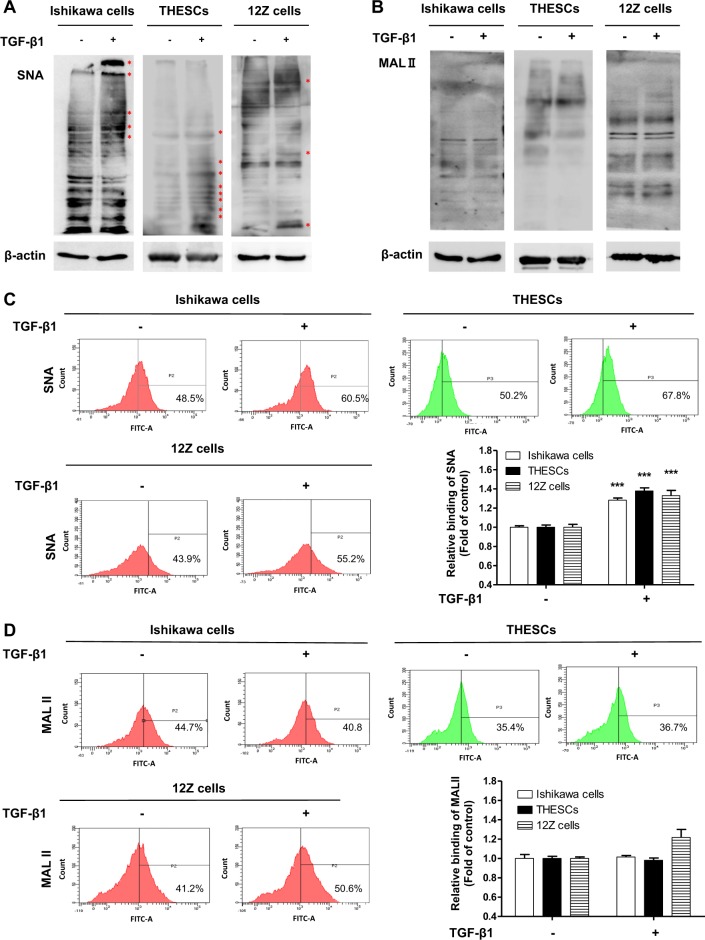
α2-6 Sialylation of endometrial cells is increased by TGF-β1 treatment Ishikawa, THESCs, or 12Z cells were incubated with TGF-β1 for 48 h. The expression of α2-3 or α2-6 sialic acid epitopes in endometrial or endometriotic cells was determined by lectin blot **a**, **b** or lectin FACS analysis **c**, **d** using biotin-labeled MAL II and SNA, respectively. Data from the lectin FACS analysis are expressed as the mean ± SD of three independent experiments. Red asterisk indicates increased protein expression in the TGF-β1-treated group compared with the control group. * *p* < 0.05 and *** *p* < 0.001 compared with the control group

Results from the lectin blot analysis showed that α2-6 sialylation was significantly increased by TGF-β1 treatment in Ishikawa, THESCs, and 12Z cells (Fig. 2a). In addition, the results of lectin FACS analysis also revealed that the expression of α2-6 sialic acid was elevated by TGF-β1 stimulation (Fig. 2c). Meanwhile, the expression of α2-3 sialic acid was not affected by exposure to TGF-β1 (Fig. 2b–d). From these results, we verified that TGF-β1 induces α2-6 sialylation in endometrial cells.

**Fig. 3:**
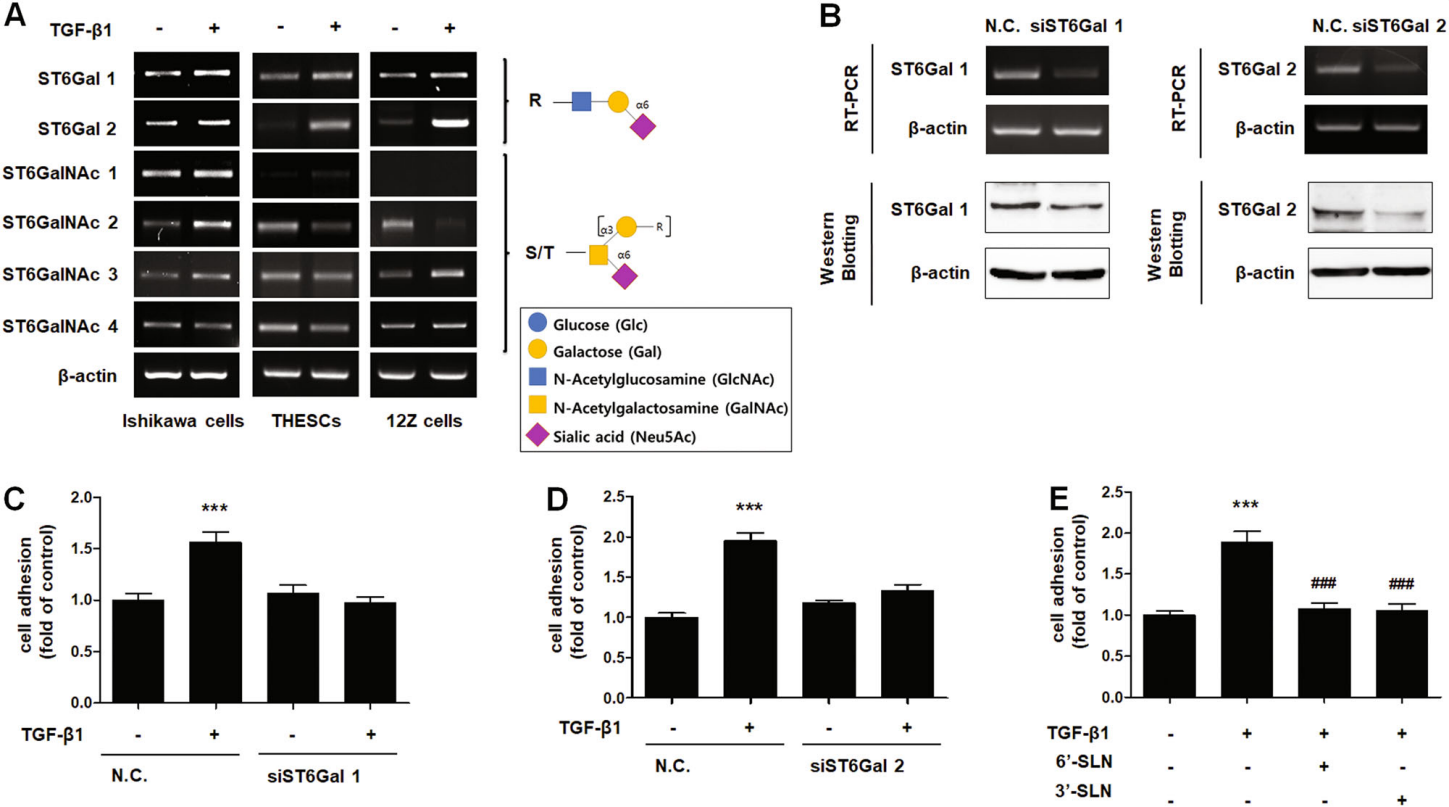
TGF-β1-induced α2-6 sialylation is mediated by increased expression of ST6Gal1 and ST6Gal2 in endometrial cells **a** Ishikawa, THESCs, and 12Z cells were treated with TGF-β1 for 24 h. Expression of α2-6 sialyltransferases was estimated by RT-PCR. **b–d** Ishikawa cells were transfected with 200 nM ST6Gal1- or ST6Gal2-targeting siRNA. **b** Expression of ST6Gal1 or ST6Gal2 in Ishikawa cells was determined by RT-PCR or western blot analysis after 24 h of incubation. **c**, **d** siRNA-transfected Ishikawa cells were cultured with TGF-β1 for 48 h, and then, transfected cells were labeled with CMFDA and added onto a Met-5A cell monolayer. The number of Ishikawa cells bound to Met-5A cells was calculated and is expressed as the fold-difference relative to the control (mean ± SD). **e** Ishikawa cells were incubated with TGF-β1 for 48 h. 3ʹ-SLN or 6ʹ-SLN were applied to treat Met-5A cells 1 h before the adhesion assay. Fluorescence-labeled Ishikawa cells were incubated at 37 °C with a Met-5A cell monolayer, and then, total number of adhered Ishikawa cells was determined. ** *p* < 0.01 and *** *p* < 0.001 compared with the control group. ^#^ *p* < 0.05 compared with the TGF-β1-treated group

There are more than 20 sialyltransferases that link sialic acid via their second carbon (C2) to the carbon atom at position C3 (α2–3 sialyltransferase) or C6 (α2-6 sialyltransferase) of Gal/GalNAc glycan or to C8 (α2-8 sialyltransferase) of another sialic acid^22^. Among α2-6 sialyltransferases, *ST6Gal1* and *ST6Gal2* gene expression was elevated by TGF-β1 treatment in THESCs, Ishikawa and 12Z cells (Fig. 3a). To elucidate their role in TGF-β1-induced endometrial cell adhesion, we abated the expression of *ST6Gal1* and *ST6Gal2* genes using siRNA targeting. As shown in Fig. 3b, the expression of ST6Gal1 and ST6Gal2 was diminished in Ishikawa cells transfected with siRNA, and the adhesion of TGF-β1-induced Ishikawa cells was significantly reduced by knockdown of *ST6Gal1* and *ST6Gal2* (Fig. 3c, d, Supplementary Figure 2b and c). However, suppressing only one (either *ST6Gal1* or *ST6Gal2*) gene decreased TGF-β1-induced adhesion of endometrial cells to the basal level. Therefore, we examined whether the effects of siRNA targeting of ST6Gal1 or ST6Gal2 interfered with the expression of the other. As shown in Supplementary Figure 2a, there was no interference between the two siRNAs. Thus, we assume that the activity of ST6Gal1 and ST6Gal2 may be related to each other. Further studies are needed to determine the functional relationship between these two genes.

ST6Gal1 and ST6Gal2 are known to increase the expression of the 6ʹ-SLN glycan structure of cell surface glycoproteins. Therefore, we incubated 6ʹ-SLN with Met-5A cells before the adhesion assay to compete with the same epitope expressed on the surface of TGF-β1-exposed endometrial cells. Adhesion of Ishikawa cells to Met-5A cells was decreased by pre-incubation with 6ʹ-SLN (Fig. 3e, Supplementary Figure 2d). Interestingly, 3ʹ-SLN treatment also decreased endometrial cell adhesion (Fig. 3e, Supplementary Figure 2c). These data suggest that receptors in Met-5A cells may interact with sialic acid linked via α2-3 or α2-6 bonds to Gal/GalNAc.

### TGF-β1 elevated the expression of sialyltransferase in endometrial cells through the TGF-βRI/SMAD2/3 signaling pathway

**Fig. 4:**
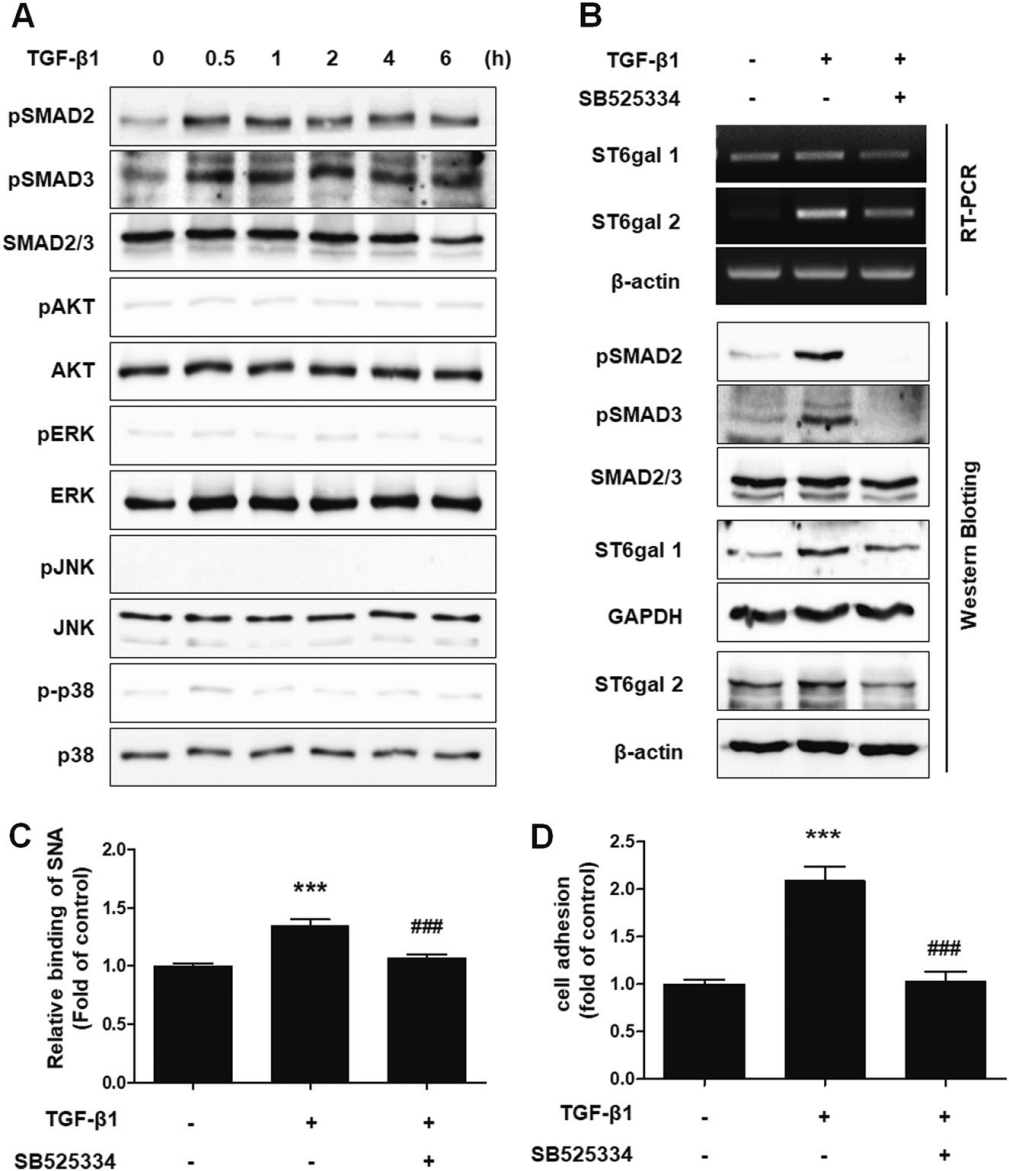
TGF-β1 induces the adhesion of endometrial cells to mesothelial cells through the TGF-βRI/SMAD2 signaling pathway **a** Ishikawa cells were cultured with TGF-β1 (10 ng/mL) according to the indicated times. Phosphorylation levels of SMAD2, SMAD3, Akt, ERK, JNK, and p38 were examined by western blot analysis. **b–d** Ishikawa cells were treated with 10 μm TGF-βRI inhibitor (SB525334) 1 h before TGF-β1 (10 ng/mL) stimulation. **b** Phosphorylation of SMAD2 and SMAD3 was determined 4 h after TGF-β1 treatment. Expression levels of ST6Gal1 and ST6Gal2 were estimated 24 h after TGF-β1 stimulation. **c** Ishikawa cells were incubated with TGF-β1 for 48 h. Using biotin-labeled SNA, α2-6 sialic acid epitopes in Ishikawa cells were analyzed via FACS using biotin-labeled SNA. **d** Tracker™ Green CMFDA-labeled Ishikawa cells were added onto Met-5A cells 48 h after TGF-β1 treatment. After incubation and washing, the number of attached Ishikawa cells was calculated and is expressed as the fold-difference relative to the control (mean ± SD). ** *p* < 0.01 for comparison between two groups. ^##^*p* < 0.01 compared with the TGF-β1-treated group

To determine the signaling pathway involved in the increased adhesion of endometrial cells induced by TGF-β1 treatment, we examined the effect of TGF-β1 treatment on mothers against decapentaplegic homolog (SMAD), mitogen-activated protein kinases and protein kinase B (Akt) signaling, which are known to be downstream signaling pathways of TGF-β1^23^. As shown in Fig. 4a, TGF-β1 significantly boosted the phosphorylation of SMAD2 and SMAD3 in Ishikawa cells 30 min after treatment. Moreover, treatment with a TGF-βRI inhibitor effectively suppressed the phosphorylation of SMAD2 and SMAD3 (Fig. 4b) and the expression of ST6Gal1, ST6Gal2, and α2-6 sialic acid epitopes (Fig. 4b, c and Supplementary Figure 3). TGF-β1-induced adhesion of Ishikawa cells to Met-5A cells was also reversed by the TGF-βRI inhibitor treatment (Fig. 4d). These results indicate that TGF-β1 increases the expression of sialyltransferase through TGF-βRI/SMAD2/3 signaling.

### 6ʹ-SLN glycan epitopes in endometrial cells interact with Siglec-9 in peritoneal mesothelial cells

**Fig. 5:**
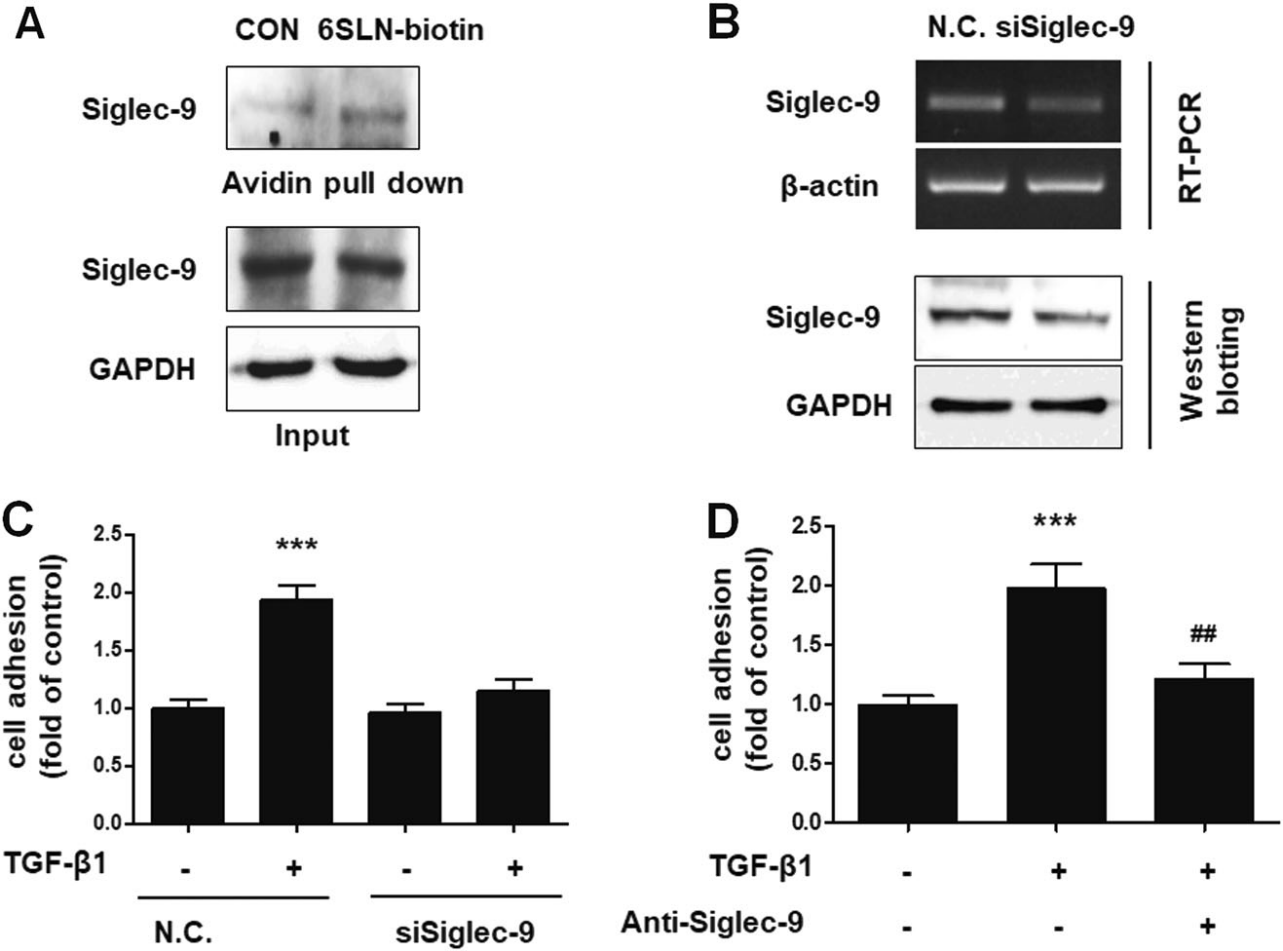
6ʹ-SLN ligands on the endometrial cells engaged Siglec-9 on Met-5A cells **a** To confirm binding between 6ʹ-SLN and Siglec-9, cell lysates from Met-5A cells were subjected to a pull-down assay using biotin-labeled 6**ʹ**-SLN and incubated with avidin beads. Proteins binding to the avidin beads were resolved and subjected to western blot analysis. **b** Met-5A cells were incubated with siRNA targeting Siglec-9 for 24 h. Expression of Siglec-9 in Met-5A cells was assessed by RT-PCR and western blot analysis. **c** siSiglec-9-transfected Met-5A cells were cultured to a confluent monolayer. Tracker™ Green CMFDA-labeled Ishikawa cells were added to the monolayer and incubated for 20 min. Adhered Ishikawa cells were manually counted, and the results are expressed as the fold-difference relative to the control (mean ± SD). **d** Ishikawa cells were treated with TGF-β1 (10 ng/mL) for 48 h. Met-5A cells were incubated with neutralizing anti-Siglec-9 antibody (2 μg/mL) 24 h before the adhesion assay. Fluorescence-labeled Ishikawa cells were added onto Met-5A cells. After 20 min of incubation and then washing, attached cells were manually counted, and the results are expressed as the fold-difference relative to the control. ** *p* < 0.01 and *** *p* < 0.001 compared with the control group. ^##^ *p* < 0.01 and ^###^ *p* < 0.001 compared with the TGF-β1-treated group

Of the 14 different mammalian Siglecs, it was previously reported that Siglec-2, Siglec-3, Siglec-5, Siglec-9, and Siglec-10 bind to 6ʹ-SLN glycan epitopes (Supplementary Table 3)^24,25^. Among these proteins, we found that only Siglec-9 was expressed in Met-5A cells (Supplementary Figure 4). To date, there have been no reports showing a Siglec-9 presence in mesothelium. Therefore, to examine if Siglec-9 can bind to 6ʹ-SLN glycan epitopes on endometrial cells, we performed a pull-down assay using 6ʹ-SLN glycan labeled with biotin. As shown in Fig. 5a, Siglec-9 expressed in Met-5A cells significantly interacted with the 6ʹ-SLN epitope. To confirm the role of Siglec-9 in enhanced endometrial–mesothelial attachment, the expression of *Siglec-9* was abated by siRNA targeting in Met-5A cells (Fig. 5b). TGF-β1-induced adhesion of Ishikawa cells to Met-5A cells was markedly attenuated after knockdown of *Siglec-9* (Fig. 5c and Supplementary Figure 5a) and after addition of a neutralizing anti-Siglec-9 antibody (Fig. 5d and Supplementary Figure 5b). These data suggest that 6ʹ-SLN glycans bind to Siglec-9 expressed on peritoneal mesothelial cells.

### 6ʹ-SLN prevents TGF-β1-induced endometriotic lesion formation in mice

**Fig. 6:**
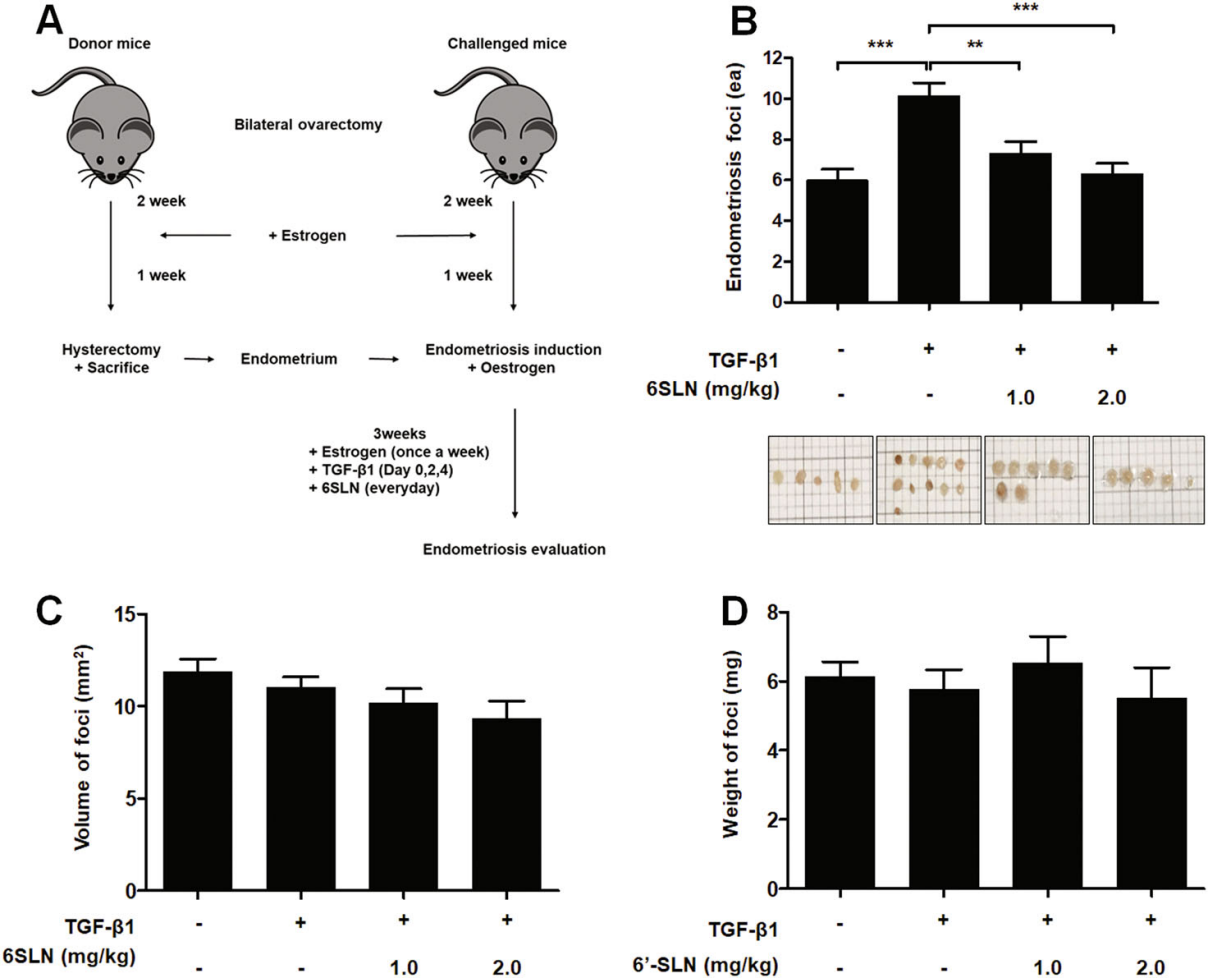
6ʹ-SLN treatment attenuates TGF-β1-induced endometriosis in C57BL/6 mice **a** Schematic diagram of the mouse endometriosis model. Endometriosis was induced at day 0. Donor and challenged mice were subjected to ovariectomy at day − 21 and injected subcutaneously with estrogens (100 mg/kg) at days − 7, 0, + 7, + 14, and + 21. Mice challenged with endometrium were inoculated intraperitoneally with 2.5 μg/kg TGF-β1 (0, + 2, + 4 days) and 6ʹ-SLN (daily). At day + 21 after endometriosis induction, challenged mice were killed, and endometriotic lesions were evaluated. **b–d** Endometriotic lesions were isolated, and the number, volume and weight of foci were calculated

**Fig. 7:**
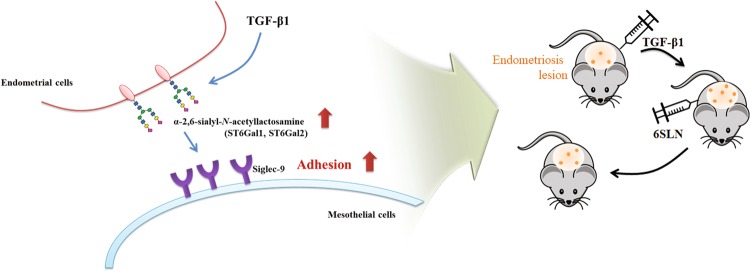
Schematic presentation of TGF-β1-induced adhesion of endometrium to mesothelium through the interaction between α2-6 sialic acid and Siglec-9 in both in vitro and in vivo endometriosis models

As shown in Fig. 6a, we established an in vivo endometriosis model according to the method described by Somigliana et al.^20^. Three weeks after endometriosis induction, mice were killed to investigate the effects of TGF-β1 and 6ʹ-SLN on the development of endometriotic lesions. TGF-β1-injected mice had more endometriotic lesions than untreated mice, whereas 6ʹ-SLN inoculation reduced the number of TGF-β1-induced total foci (Fig. 6b and Supplementary Figure 6). No significant difference was observed in the volume and weight of endometriotic lesions between the groups of mice (Fig. 6c, d). These results indicated that increased formation of endometriotic lesions owing to TGF-β1 can be prevented by using 6ʹ-SLN to inhibit sialic acid binding (Fig. 7).

## Discussion

Glycosylation is involved in diverse pathophysiological processes and contributes to the development of various diseases, such as cancer, diabetes, rheumatoid arthritis, and immunological disorders^26–28^. Particularly, aberrant expression of terminal sialic acid has been detected in various carcinoma cells, especially those that are associated with adhesion and invasion of tumor cells^29,30^. However, the effect of sialic acid glycan epitopes on the development of endometriosis is not as investigated as their effect on cancer. Previous studies have verified that glycoproteins, including carbohydrate antigen (CA) 125, CA19-9, biglycans, and *N*-glycans, are elevated in the serum or peritoneal fluid of endometriosis patients^14–16^. It has also been reported that the endometrium of women with endometriosis expresses more glycans than that of normal women, and endometriotic tissues showed increased glycan expression in a baboon model of endometriosis^31,32^. Rodgers et al.^17^ reported that inhibition of CD44 glycosylation decreases the adhesion of endometrial cells to the mesothelium. Although there are various theories regarding the pathogenesis of endometriosis—retrograde menstruation, coelomic metaplasia, altered immunity, and genetic basis—the attachment of endometrial cells is known as a pivotal step in the onset of endometriosis^2,33^. In the present study, we showed that enhanced expression of sialylated glycan increases adhesion of endometrial cells to the peritoneal mesothelium. Subsequently, induced mesothelial adhesion of endometrial cells and tissues was efficiently prevented by the application of a glycan epitope.

TGF-β is a multifunctional growth factor involved in regulating cell proliferation, differentiation, and cell–cell interaction^7,34^. It has been reported that TGF-β1 expression is significantly higher than normal in serum, peritoneal fluid, and cyst tissues of endometriosis patients and that TGF-β1 plays a pivotal role in the progression of endometriosis^8,35–37^. In the present study, TGF-β1 enhanced adhesion of endometrial cells to peritoneal mesothelial cells through induction of endometrial cell sialylation. IL-1β, IL-6, and IL-8 also increased the adhesion of endometrial cells. However, neuraminidase treatment could not sufficiently restore cytokine-induced adhesion of endometrial cells to the mesothelium. Therefore, there may be other factors responsible for the increased cell adhesion induced by IL-1β, IL-6, or IL-8 treatment aside from sialylation changes.

Sialic acid, a terminal monosaccharide of glyco-conjugates, is linked via an α2-3 or α2-6 bond to Gal/GalNAc or an α2-8 bond to another sialic acid in proteins^22^. According to the lectin blot and lectin FACS analysis results, α2-6 sialylation increased after TGF-β1 stimulation. Sialyltransferase, an enzyme that transfers sialic acid to oligosaccharides, is responsible for the degree of sialylation on cell surfaces^38^. Therefore, we examined whether the expression of sialyltransferase was altered by TGF-β1 treatment. Among the 20 human sialyltransferases, only two, ST6Gal1 and ST6Gal2, were more highly expressed in TGF-β1-treated endometrial cells than in untreated cells. Both enzymes are part of the α2-6 sialyltransferase family and transfer sialic acid residues to Galβ1-4GlcNAc disaccharide sequences using an α2-6 linkage to form a 6ʹ-SLN glycan structure^39^. Although *ST6Gal1* is detected in almost every tissue, *ST6Gal2* shows a restricted, tissue-specific expression pattern, mainly in brain and fetal tissues and is expressed to a lesser degree in testes, thyroid glands, lungs, and other tissues. Previous studies have shown that ST6Gal1 is aberrantly expressed in various cancers, such as colon, breast, and pancreatic cancer. In addition, altered expression of ST6Gal1 is associated with adhesion and metastasis of cancer cells^30,40,41^. However, pathological alterations in ST6Gal2 expression are not well understood^39,40,42^. The expression of *ST6Gal1* and *ST6Gal2* is regulated at the transcriptional level through specific promoters. Three different promoters have been reported to adjust *ST6Gal1* expression^43^. In particular, Sp1 and SMAD binding sites within the *ST6Gal1* promotor were involved in the upregulation of ST6Gal1 during TGF-β1-induced epithelial–mesenchymal transition in mouse epithelial GE11 cells^44^. Lehoux et al.^39^ suggested that Sp1-binding sites are also essential for transcription of *ST6Gal2* in human neuronal cells, but regulation of *ST6Gal2* under the stimulation of TGF-β1 has not yet been reported. In this study, the expression of both *ST6Gal1* and *ST6Gal2* increased after TGF-β1 treatment, and consequently, both are likely involved in the adhesion of endometrial cells through increasing sialylation levels. We assumed that TGF-β1 increased the expression of *ST6Gal1* and *ST6Gal2* via a similar mechanism in endometrial cells.

Next, we confirmed the signaling pathways engaged in glycosylation-mediated adhesion of endometrial cells after TGF-β1 treatment. In the canonical pathway, TGF-β1 signaling is initiated by the formation of a serine/threonine kinase receptor complex, and then, phosphorylated-SMAD2/3 protein binds to SMAD4 and is translocated to the nucleus, where it binds to DNA^23^. However, it has been reported that SMAD-independent signaling pathways, such as extracellular signal-regulated kinase, p38, c-Jun N-terminal kinase, and Akt, also participate in TGF-β signaling^23,45^. In our experiments, only SMAD-dependent signaling responded to TGF-β1 stimulation in endometrial cells.

To identify the receptor for sialic acid-conjugated glycan structures, we examined the expression and function of Siglecs in mesothelial cells. Siglecs are cell surface proteins that consist of an immunoglobulin-like domain with a site that binds to sialic acid-containing glycans, a trans-membrane domain, and a cytosolic domain that contains a signaling motif^46^. There are, at present, 14 known Siglecs in humans and nine Siglecs in mice that are primarily expressed on immune cell surfaces^25^. Siglecs have been thought to regulate the functions of innate and adaptive immune cells and promote cell–cell interaction through sialic acid recognition^25,47,48^. Previously, studies have shown that Siglecs are specific receptors for sialylated glycans in leukocytes and carcinoma cells and that they regulate the adhesion of these cells to the endothelium for migration^49–51^.

Siglecs are mainly expressed by cells of the hematopoietic, immune, and nervous systems, and expression in other tissues is rarely reported^52^. In the present study, we demonstrated that Siglec-9 is expressed in peritoneal mesothelial cells and interacts with sialylated glycan epitopes in endometrial cells. To the best of our knowledge, this is the first study showing Siglec-9 expression in the mesothelium. Furthermore, inhibiting the function of Siglec-9 reduces the interaction between the endometrium and mesothelium. Siglec-9 is predominantly expressed on monocyte and neutrophil surfaces, and both cells are descendants of hematopoietic stem cells^53^. The embryological origin of both hematopoietic stem cells and mesothelium is the embryonic mesoderm cell layer^54^. Therefore, we theorize that the expression of Siglec-9 in peritoneal mesothelial cells may be related to an embryological origin.

Previous studies have reported that genetic deficiency of TGF-β1 or pharmacological inhibition of TGF-βR suppresses endometriotic lesion development in a mouse endometriosis model^55,56^. However, in women with endometriosis, the concentration of TGF-β1 is enhanced in the peritoneal cavity, which plays an important role in the establishment of endometriosis^36^. In this study, we demonstrated that TGF-β1 injections increased the formation of endometriotic foci in a murine endometriosis model. As far as we know, this is the first report showing in an in vivo model that the formation of endometriotic lesions is induced by TGF-β1 stimulation. Moreover, intraperitoneal injection of 6ʹ-SLN glycan ameliorates the development of TGF-β1-induced endometriotic lesions. 6ʹ-SLN is an oligosaccharide component possessing various structures that is present in human milk^57^. Previous studies have reported that human milk oligosaccharides have various biological functions, such as inhibition of microbial adhesion, prevention of pathogen attachment, and immune system regulation, as well as anti-inflammatory and anti-angiogenic properties^58–60^. Therefore, 6ʹ-SLN could be a potentially useful therapeutic candidate for endometriosis treatment by preventing the attachment of endometrial tissues onto the peritoneum.

In the present study, we have demonstrated for the first time that TGF-β1 increases α2-6 sialylation of endometrial cells through the induction of ST6Gal1 and ST6Gal2 sialyltransferase expression and consequently increased endometrial–mesothelial adhesion. We also discovered that sialylated glycan epitopes bind to Siglec-9 expressed on mesothelial cell surfaces. In addition, application of 6ʹ-SLN glycan was shown to prevent adhesion of endometrial cells and formation of endometriotic lesions in an in vivo endometriosis model. Based on these results, we suggest that altered sialylation of the endometrium is a significant factor in endometriosis development.

## Electronic supplementary material

Supplementary data
