# Supplementary material for: Increased α2-6 sialylation of endometrial cells contributes to the development of endometriosis
Source: Exp Mol Med. 2018 Dec 12;50(12):164. doi: 10.1038/s12276-018-0167-1 (PMC6290765; doi:10.1038/s12276-018-0167-1)
Supplement: Supplementary file 1 — Supplementary data [file 12276_2018_167_MOESM1_ESM.docx]

**Supplementary Table 1. Information of antibodies used in this study.**

| Antibody name | Company | Catalog number | Dilution for Western blot analysis |
| --- | --- | --- | --- |
| Anti-ST6Gal1 antibody | Signalway Antibody | #34601 | 1:500 |
| Anti-ST6Gal2 antibody | Atlas Antibodies | HPA014459 | 1:500 |
| Anti-pSMAD2 antibody | Cell Signaling Technology | #3101 | 1:500 |
| Anti-pSMAD3 antibody | Cell Signaling Technology | #9520 | 1:500 |
| Anti-SMAD2/3 antibody | Santa Cruz Biotechnology | sc133098 | 1:100 |
| Anti-pAKT antibody | Abcam | ab81283 | 1:1000 |
| Anti-AKT antibody | Abcam | ab32505 | 1:1000 |
| Anti-pERK antibody | Santa Cruz Biotechnology | sc-7383 | 1:100 |
| Anti-ERK antibody | Santa Cruz Biotechnology | sc-154 | 1:100 |
| Anti-pJNK antibody | Cell Signaling Technology | #9255 | 1:1000 |
| Anti-JNK antibody | Cell Signaling Technology | #9253 | 1:1000 |
| Anti-pp38 antibody | Cell Signaling Technology | #9211 | 1:1000 |
| Anti-p38 antibody | Santa Cruz Biotechnology | sc-535 | 1:100 |
| Anti-Siglec-9 antibody | Nobus Biologicals | NBP1-32542 | 1:500 |
| Goat anti-Rabbit IgG Secondary Antibody | Thermo Fisher Scientific | 31460 | 1:4000 |
| Goat anti-Mouse IgG Secondary Antibody | Thermo Fisher Scientific | 31430 | 1:4000 |

**Supplementary Table 2. Primer sequence used in this study.**

| Gene | Primer sequence | Size (bp) |
| --- | --- | --- |
| *ST6Gal1* | F: CTTCTGTTTGCAGTCATCTGTGTG | 471 |
|  | R: AATGCTCTCCTTGGGCAGATAACCC |  |
| *ST6Gal2* | F: TTCCAAAATGCTGAACCCGC | 286 |
|  | R: AGAGTTGAGGATTGCGCCTG |  |
| *ST6GalNAc1* | F: GTCCAGTGGTCCTTGCTTCTGGCTG | 459 |
|  | R: ATTTCCTTGGGTCGTCTTTGTGTCC |  |
| *ST6GalNAc2* | F: GGCTCCTCTTTGCCCTGTACTTCTC | 545 |
|  | R: GTTCACAGTGAAACCATAGAAGGAA |  |
| *ST6GalNAc3* | F: CTGTGATTGCTGTGAGCTTCATAG | 380 |
|  | R: AAGAGGAACGCTGGTATGGGACACA |  |
| *ST6GalNAc4* | F: CGGCTCGTGCTCATCATCCTGTGCT | 503 |
|  | R: GTGAGCTGCAGCAGCGTGCGGTAGG |  |
| *β-actin* | F: CAAGAGATGGCCACGGCTGCT | 275 |
|  | R: TCCTTCTGCATCCTGTCGGCA |  |
| *Siglec-2* | F: TTTGCTCTCAGATGCTGCCA | 564 |
|  | R: CAGCAAGCAGGTCAGAGTGA |  |
| *Siglec-3* | F: AGTGACGGTACAGGAGGGTT | 375 |
|  | R: AGAGTGCCAGGGATGAGGAT |  |
| *Siglec-5* | F: TCTTCTTTTTAATAGTGAAAGCCCG | 235 |
|  | R: TCCTTAGGCTCCCTCGACTT |  |
| *Siglec-9* | F: CCTCTAAGTCTTGAGCCCGC | 503 |
|  | R: ACATTCACAGAGAGCCGGTG |  |
| *Sigelc-10* | F: ATTTCATGCATCAGGCCCCA | 584 |
|  | R: CGGGGATGTAGACATCAGGC |  |

**Supplementary Table 3. Relative affinities of Siglecs to sialyllactosamines.**

| Siglecs | Glycan structure | |
| --- | --- | --- |
|  | 3ʹ-SLN | 6ʹ-SLN |
| Siglec-1 |  |  |
| Siglec-2 |  | + |
| Siglec-3 |  | + |
| Siglec-4 | + |  |
| Siglec-5 | + | + |
| Siglec-6 |  |  |
| Siglec-7 | + |  |
| Siglec-8 | + |  |
| Siglec-9 | + | + |
| Siglec-10 | + | + |
| Siglec-11 |  |  |
| Siglec-12 |  |  |
| Siglec-13 |  |  |
| Siglec-14 | + |  |

The table shows affinities of Siglecs to 3ʹ-SLN or 6ʹ-SLN glycan epitopes.

+: detectable binding


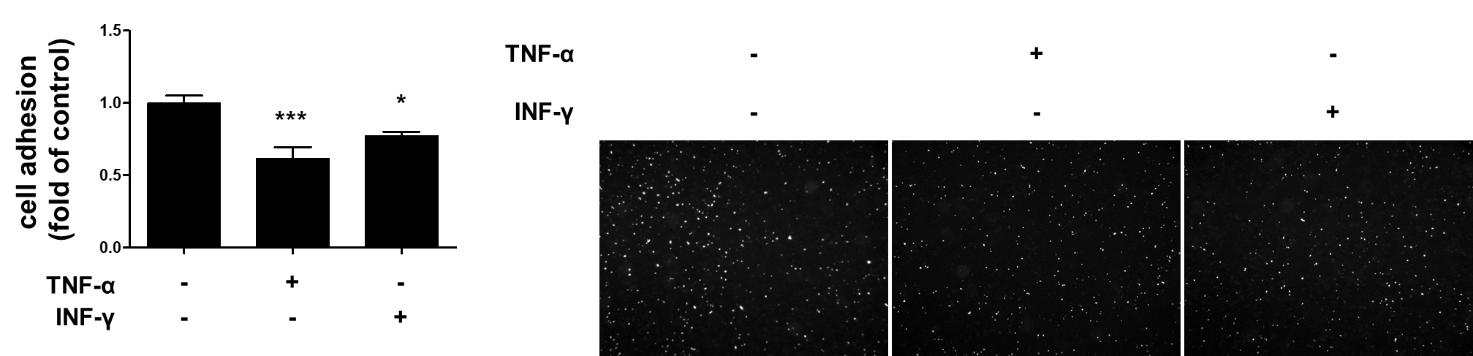


**Supplementary Figure 1 Adhesion of Ishikawa cells to Met-5A cells after treatment with TNF-α or INF-γ.** Ishikawa cells were treated with TNF-α or INF-γ (10 ng/mL) for 48 h. Fluorescence-labeled Ishikawa cells were added onto Met-5A cells, and the attached cells were counted. * *p* < 0.05 and ** *p* < 0.01 when compared to control group.


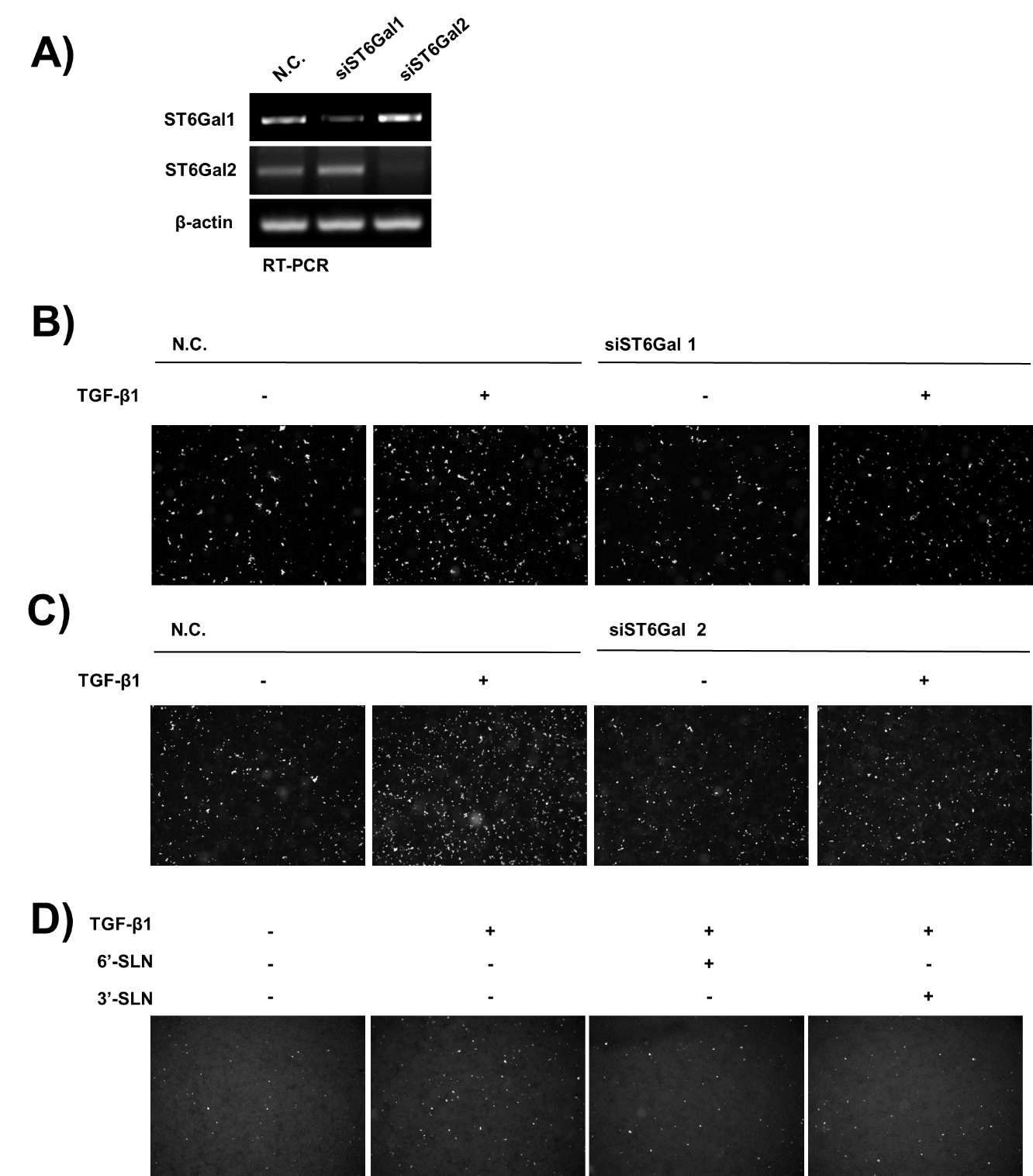


**Supplementary Figure 2 TGF-β1-induced adhesion of Ishikawa cells was mediated by ST6Gal1 and ST6Gal2. (A)** Ishikawa cells were transfected with ST6Gal1 or ST6Gal2 targeting siRNA, and the expression of ST6Gal1 or ST6Gal2 was determined by RT-PCR. **(B, C)** Adhesion of ST6Gal1- or ST6Gal2-abated endometrial cells to the peritoneum. **(D)** Adhesion of endometrial cells to peritoneal cells after SLN treatment.


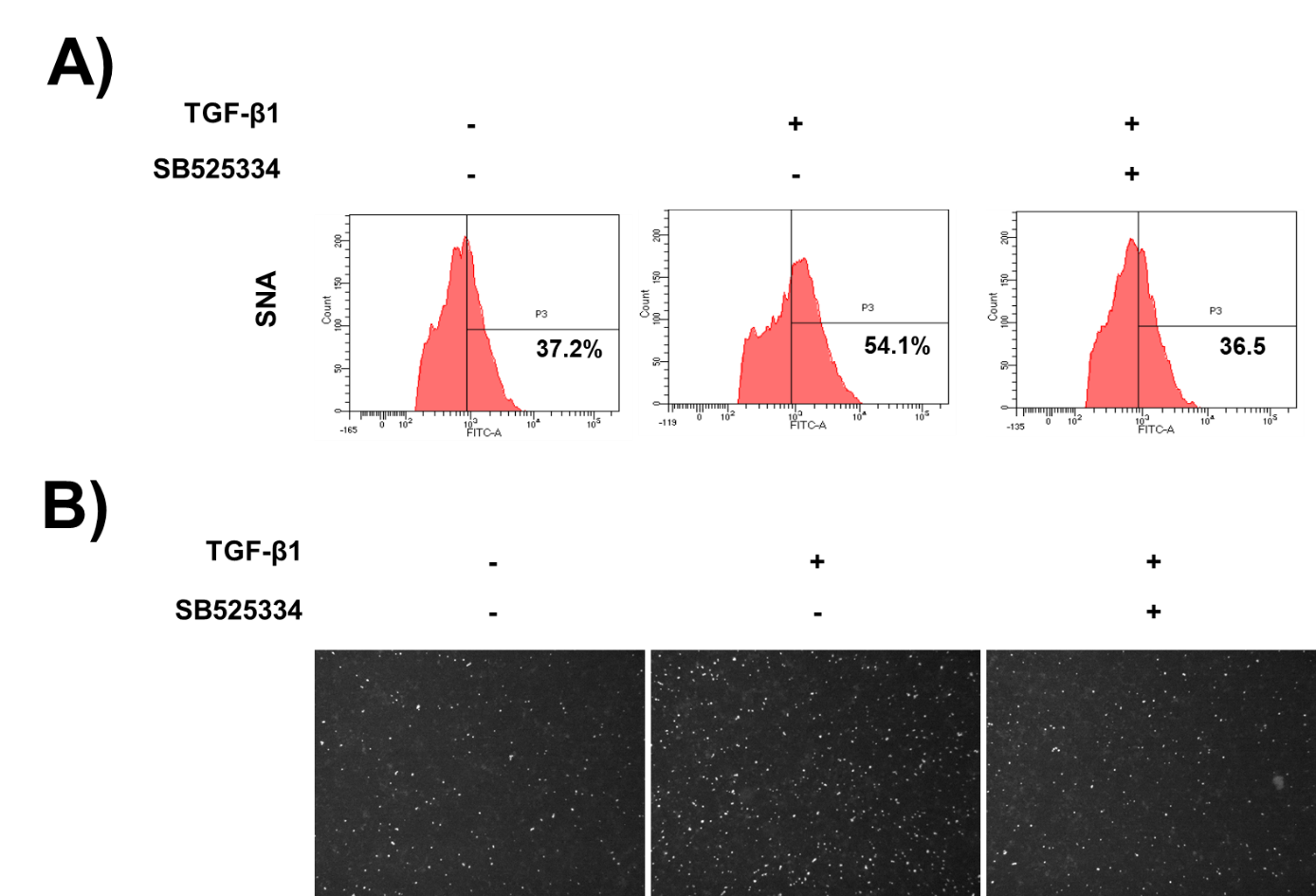


**Supplementary Figure 3 The effect of TGF-βRI inhibitor on α2-6 sialic acid expression and Ishikawa cell adhesion.** **(A)** Expression of sialic acid after TGF-β1 and TGF-βRI inhibitor (SB525334) treatments in Ishikawa cells. **(B)** Adhesion of Ishikawa cells onto Met-5A cells after addition of TGF-β1 and TGF-βRI inhibitor.


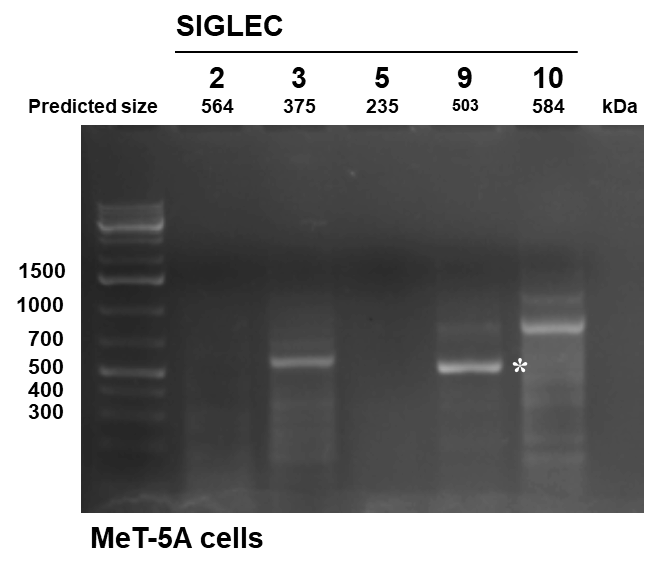


**Supplementary Figure 4 Expression of Siglecs that bind to 6ʹ-SLN glycans in Met-5A cells.** Total RNA of Met-5A cells were extracted and the expression of Siglecs was determined by RT-PCR.


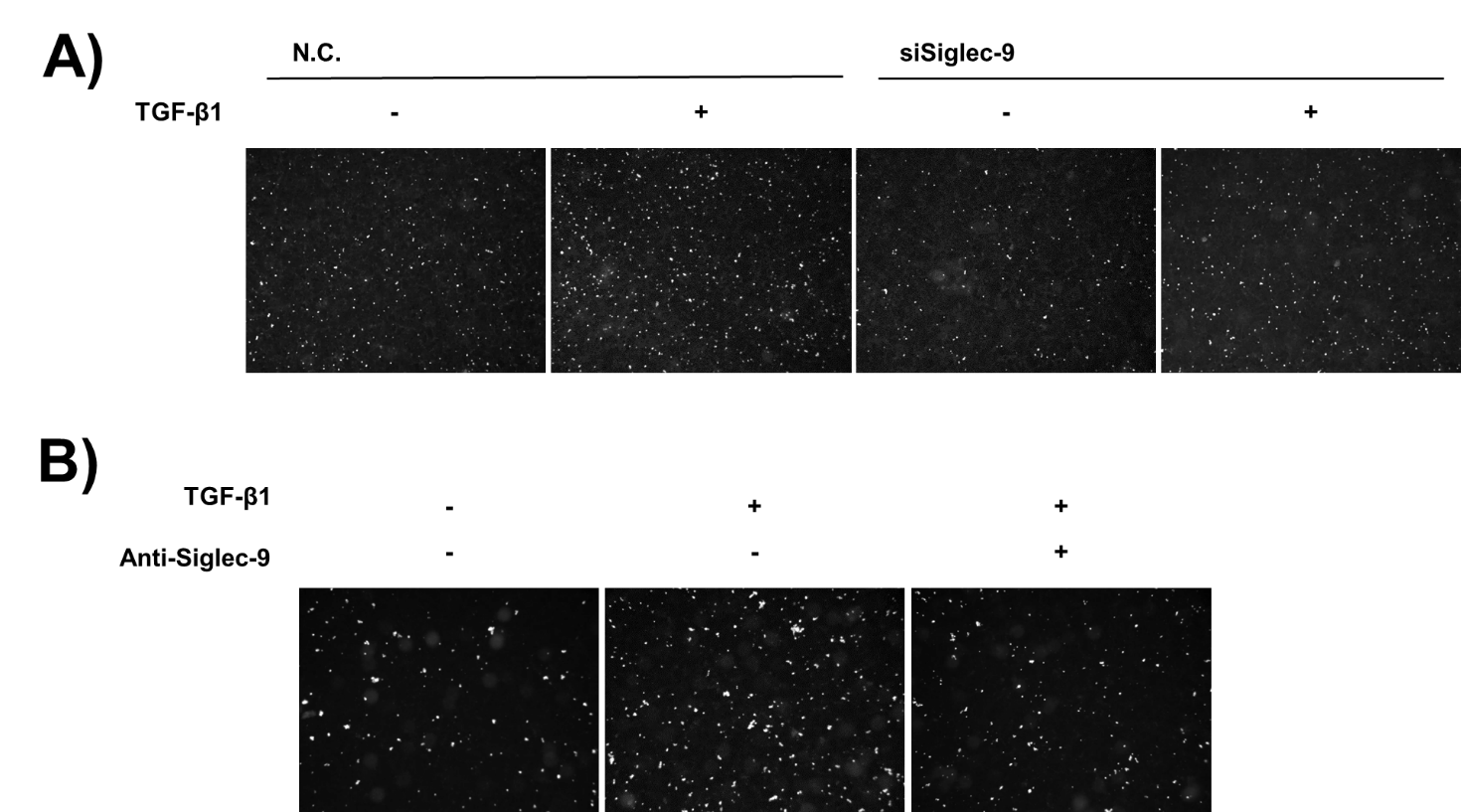


**Supplementary Figure 5 Effect of Siglec-9 on TGF-β1 induced Ishikawa cell adhesion. (A)** Adhesion of Ishikawa cells to Siglec-9 abated Met-5A cells. **(B)** Interaction between Ishikawa cells and Met-5A cells after Siglec-9 neutralizing antibody treatment.


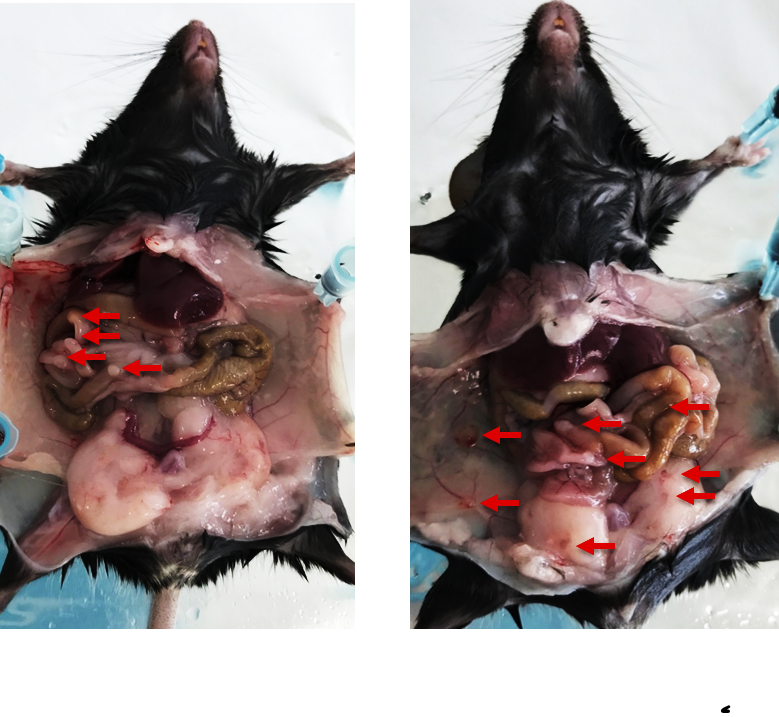


**Supplementary Figure 6 Endometriotic lesion development in a mouse endometriosis model.** Mice were sacrificed, and a laparotomy was performed to verify the endometriotic lesion formation, at 3 weeks after endometriosis induction. Endometriosis foci (red arrows) were attached to the peritoneum, omentum and fat layers in the uterine cavity.
